# Birds That Don't Exist: Niche Pre‐Emption as a Constraint on Morphological Evolution in the Passeroidea

**DOI:** 10.1111/ele.70320

**Published:** 2026-02-01

**Authors:** Stephanie Y. Chia, Anshuman Swain, Nathaniel Josephs, Lizhen Lin, William F. Fagan

**Affiliations:** ^1^ Department of Biology University of Maryland College Park Maryland USA; ^2^ Department of Ecology and Evolutionary Biology University of Michigan Ann Arbor Michigan USA; ^3^ Museum of Paleontology University of Michigan Ann Arbor Michigan USA; ^4^ Department of Statistics North Carolina State University Raleigh North Carolina USA; ^5^ Department of Mathematics University of Maryland College Park Maryland USA

**Keywords:** ancestral state reconstruction, ecological niche, macroecology, macroevolution, morphological evolution, niche preemption, Passeroidea, persistent homology, topological data analysis, trait space

## Abstract

Understanding why some viable body forms never evolve can reveal how ecological and evolutionary forces shape biodiversity. We investigate this question in the Passeroidea, a large group of songbirds, by analysing their morphological trait space using topological data analysis and ancestral state reconstruction. We identify a persistent morphological gap densely surrounded by extant species but unoccupied throughout passeroid diversification. The gap patterns deviate from stochastic expectations and show no evidence of past occupation, rendering undirected trait evolution and extinction unlikely. Similar morphologies exist in other bird lineages, ruling out intrinsic constraints or niche absence. Geographic distributions and traits of passeroids versus non‐passeroid gap occupants point to competitive exclusion as the plausible explanation: early‐colonising territorial specialists outside the Passeroidea may have preemptively occupied key habitats, limiting evolutionary opportunities for later‐arriving lineages. We demonstrate how historical contingency can shape macroevolutionary outcomes and introduce a generalizable framework for investigating structural gaps in trait evolution.

## Graphical abstract

We use topological data analysis to reveal a persistent morphological gap in a major group of songbirds (superfamily Passeroidea). The gap remained unoccupied for millions of years, even though nearby morphologies are common and the same body form exists in other groups of birds. Our results suggest that competitive exclusion has constrained the evolution of this missing form.

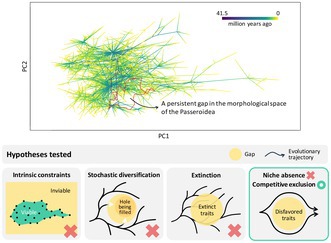

## 1 Introduction

Birds, with over ten thousand species, exhibit remarkable morphological diversity. This variation in body form underlies their ecological success, enabling species to exploit a wide range of environments and resources. Morphological traits are tightly linked to ecological function. For example, beak shapes reflect feeding specialisation such as seed‐cracking or insect‐catching (Herrel et al. 2005; Lack 1983), wing and tail morphologies influence flight style and migratory ability (Norberg 1990), leg length relates to habitat use (from long‐legged waders in aquatic habitats to short‐legged aerial insectivores that minimise drag; Zeffer et al. 2003), and body mass affects thermoregulation, starvation resistance, and predator–prey dynamics (Peters 1986).

Together, these traits define a multidimensional morphological trait space, or morphospace, wherein each species occupies a unique position corresponding to its ecological niche (Hutchinson 1957; Pigot et al. 2020). Unoccupied regions in this morphospace, so‐called morphological gaps, may reflect nonviable trait combinations, ecological niches that do not exist or are biophysically or biogeographically inaccessible or viable strategies that have not yet evolved. Identifying and understanding these gaps offers insight into the ecological and evolutionary forces that shape the limits of biodiversity.

Detecting such gaps in high‐dimensional space is challenging, especially when these gaps lie within otherwise densely occupied regions. Such ‘holes’ can be obscured by dimensionality reduction techniques commonly used in trait‐based analysis. To address this, Blonder (2016) introduced a probabilistic framework to detect holes in high‐dimensional hypervolumes by comparing observed trait distributions to convex or maximal expectations. This kernel density‐based approach opened new avenues for exploring trait space, including studies of extinction risk in birds (Ali et al. 2023). However, it requires selecting a bandwidth and may be sensitive to uneven trait distributions, which are common for data in morphospaces.

As an alternative, we explore persistent homology, a method from topological data analysis that detects topological features in multidimensional point clouds (Edelsbrunner et al. 2002) without assuming smoothness or evenness in data distribution. It works by progressively connecting nearby species in the trait space and detecting loops that persist across a range of distance thresholds, thereby revealing robust gaps. Imagine a flooded landscape (morphospace) with many mountain peaks (species). As water level gradually drops (increasing distance threshold), peaks connect into ridges; when ridges form a loop, a lake appears (birth of a morphological gap). As water level continues to drop, the lake eventually disappears as the terrain fills in (death of the gap). The longer the lake persists across water levels, the more robust the gap is. Although the potential of persistent homology for studying ecological niche hypervolumes and trait distributions has been noted (Conceição and Morimoto 2022), it remains largely unexplored.

We apply persistent homology to examine the structure and temporal dynamics of the morphospace in the superfamily Passeroidea, a large clade of songbirds, as a case study. By combining persistent homology with ancestral state reconstruction, we detect and track the existence of morphological gaps through evolutionary time. Specifically, we ask: (1) Are there combinations of morphological traits that do not exist among extant passeroids? (2) If so, what ecological or evolutionary processes explain these absences?

**FIGURE 1:**
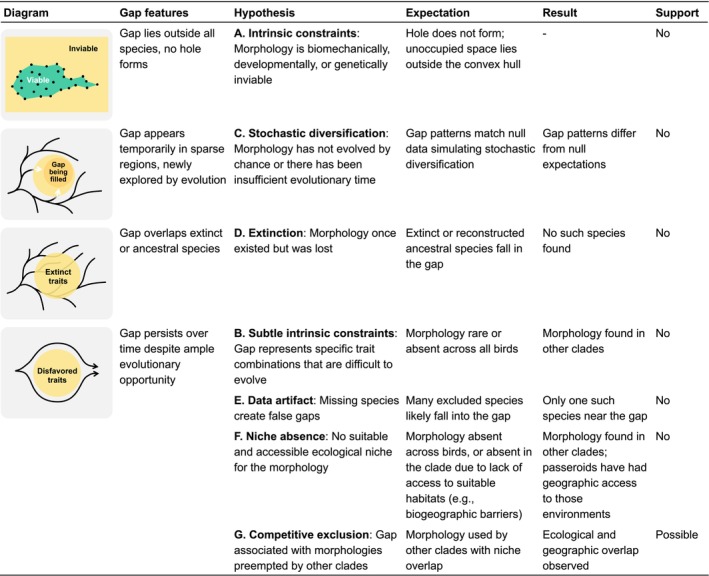
Conceptual framework and evaluation of hypotheses for the presence of a morphological gap in the Passeroidea trait space. Diagrams illustrate gap features associated with each hypothesis, with yellow areas indicating unoccupied regions of morphospace and black curves showing evolutionary trajectories through morphospace. Hypotheses D through G share the same gap diagram. The expectation, observed result and level of support for each hypothesis are summarised.

To address these questions, we consider several hypotheses (Figure 1). Morphological gaps may reflect intrinsic constraints, such as biomechanical, developmental, or genetic limitations that make certain trait combinations inviable, either lying outside of the observed range of extant species (Hypothesis A), or due to more subtle limitations within the realised morphospace (Hypothesis B). Gaps might also emerge as a stochastic outcome of trait diversification, reflecting viable trait combinations that have not evolved simply due to chance or insufficient evolutionary time (Hypothesis C). Other possibilities include extinction of lineages that once occupied the gap (Hypothesis D), artefacts of missing data (Hypothesis E), or ecological mismatch whereby suitable niches for the morphology do not exist or are inaccessible (Hypothesis F). Lastly, a morphology might be viable and realised in other clades but absent from the focal clade due to competitive exclusion (Hypothesis G). We assess these seven possibilities by examining the evolutionary persistence of morphological gaps, their positions in morphospace, evidence of historical occupation by ancestral or extinct species, and the ecological and phylogenetic context of species that are morphologically similar.

## 2 Materials and Methods

### 2.1 Morphological and Ecological Traits

We analyze the superfamily Passeroidea as a case study to explore morphological macroevolution. This large monophyletic clade (Mackiewicz et al. 2019) within the passerines includes approximately 1400 species, including sparrows, finches, tanagers, pipits and many types of warblers. Members of the Passeroidea share relatively similar morphology, resulting in a densely populated morphospace that is well suited for detecting robust topological features.

We use 10 morphological traits obtained from the AVONET dataset (Tobias et al. 2022): beak length from tip to skull along culmen, beak length from tip to anterior edge of nares, beak width, beak depth, tarsus length, wing length (carpal joint to wingtip), secondary length (carpal joint to tip of outermost secondary), tail length, hand‐wing index and body mass. Kipp's distance was excluded due to redundancy with other wing measurements. These 10 traits provide standardised, high‐coverage measurements of external morphology relevant to avian form and function, making them suitable for large‐scale comparisons across species. We excluded 40 extant species that had at least one morphological trait (excluding body mass) referenced from a closely related species, resulting in a final dataset of 1378 extant species. All traits were log‐transformed (except the hand‐wing index) and standardised to a mean of zero and standard deviation of one. We applied principal component analysis (PCA) and retained the first four principal components (PCs), which together explained 95% of the total variance. High‐order PCs (< 5% of variance) were excluded due to computational constraints and their minimal contribution to the overall trait variation, which was unlikely to affect the main morphospace structure.

Morphological data for 60 recently extinct passeroid species (i.e., extinct within the past ~130,000 years) were obtained from the AVOTREX dataset (Sayol et al. 2023). Earlier extinct species were not included due to the lack of comparable morphological data. Secondary length was calculated as the difference between wing length and Kipp's distance, whereas the hand‐wing index was calculated as Kipp's distance divided by wing length. These extinct species were projected onto the morphospace defined by extant species' PCs.

Trophic niche, primary lifestyle, and breeding range centroid and range size were also obtained from the AVONET dataset (Tobias et al. 2022). Territoriality was sourced from Tobias et al. (2016). BirdTree taxonomy (Jetz et al. 2012) was adopted to compile all trait data.

### 2.2 Ancestral State Reconstruction and Trait Evolution Simulations

Phylogenetic tree data were retrieved from BirdTree.org (Jetz et al. 2012), which provides a posterior distribution of global avian phylogenies generated under a Bayesian framework by combining multilocus molecular sequence data with higher‐level phylogenomic constraints from Hackett et al. (2008), incorporating fossil calibrations and relaxed‐clock models to estimate divergence times. From the Hackett backbone trees, we randomly sampled 1000 trees and generated a consensus tree using majority rule topology and least squares edge length estimation.

Ancestral state reconstruction for each PC was performed under a Brownian Motion model using generalised least squares (Figure S1). Although Brownian motion assumes unconstrained trait evolution, performing the reconstruction in the PC space, rather than the original trait space, allows it to occur within an empirically derived morphospace that captures the main patterns of trait covariation. The resulting root values and evolutionary rate (*σ* ^2^) were used to generate 10 null trait datasets under Brownian motion using the same consensus tree. Because both the empirical reconstructions and null simulations were conducted under the same model assumptions, differences in gap properties between them reflect deviations from a shared null expectation, minimising potential model‐specific effects. Trait values for all datasets were linearly interpolated at 1‐million‐year intervals based on the tip (present‐day trait values) and ancestral node values.

All phylogenetic analyses and simulations were performed in R using the package *phytools* (Revell 2024).

### 2.3 Gap Identification and Characterisation

At each time slice, we applied persistent homology to detect morphological gaps in the four‐dimensional morphospace defined by the first four PCs. Persistent homology is a topological data analysis tool that can identify features such as holes in multidimensional point clouds (Otter et al. 2017). We used Vietoris‐Rips filtration based on Euclidean distance to detect loop‐like structures (H_1_ features), which we interpret as morphological gaps. For each detected gap, we extracted the coordinates of the points forming the loop for downstream analysis. Computations of persistent homology were performed using the *Dionysus* C++ library via the R package *TDA* (Fasy et al. 2025).

Each gap's topological persistence (i.e., the difference between ‘gap birth’ and ‘gap death’ values) describes how long (across what range of distance scales) a gap remains detectable as nearby species are progressively connected in the morphospace. For example, if a gap appears when points within a distance of 0.5 are connected (‘birth’) and disappears when points within a distance of 1.0 are connected (‘death’), its persistence would be 0.5. This value therefore quantifies the robustness of the structure across distance scales in the morphospace, with higher topological persistence indicating stronger and more stable topological structure.

We also computed the gap centroid as the mean location of its vertices, gap size as the mean distance from the centroid to its vertices, and sparsity as the mean distance from the centroid to the nearest 5% of species (69 of 1378 data points), serving as an intuitive, distance‐based measure of local density. All these metrics are in PC distance units (dimensionless). To account for the irregular shapes of topological gaps, we defined a species as being ‘within’ or ‘near’ a gap based on scaled distances from the gap centroid, using half and full gap sizes as heuristic thresholds, respectively.

We extracted notable gap structures with topological persistence > 0.4 (PC unit), a heuristic threshold chosen based on the empirical distribution of persistence values (Figure 5A). Only about 0.5% of all detected gaps exceeded this value, allowing us to focus on the most robust structures. Gaps between consecutive time slices were linked into gap series if the centroids were within 1 unit. For each gap series, we calculated evolutionary lifespan (duration of gap persistence in millions of years), average size and average sparsity. The analysis was limited to the last 10 million years, beyond which the number of reconstructed ancestral nodes becomes too sparse to reliably characterise topological structure.

## 3 Results

### 3.1 Identification of Morphological Gap in Passeroidea

**FIGURE 2:**
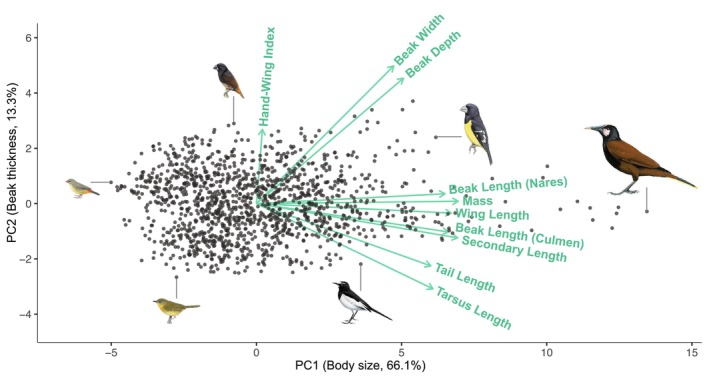
Morphological trait space of 1378 extant Passeroidea species. Each point represents a species positioned by its scores on the first two principal components, which together explain 79.4% of the total morphological variance. PC1 primarily reflects body size, and PC2 corresponds to beak thickness. Green arrows indicate the loadings of the original morphological traits on the PC axes. Species illustrations (from left to right: Zebra Waxbill, Hooded Yellowthroat, Bismarck Munia, Japanese Wagtail, Spot‐winged Grosbeak and Baudo Oropendola) are from Birds of the World, Cornell Lab of Ornithology.

Our analysis included 1378 extant species (97.2%) in the superfamily Passeroidea. The first four principal components explain 95% of total morphological variance and primarily capture body size (PC1), beak thickness (PC2), hand‐wing index (PC3) and beak length (PC4) (Figure 2, Table S1).

**FIGURE 3:**
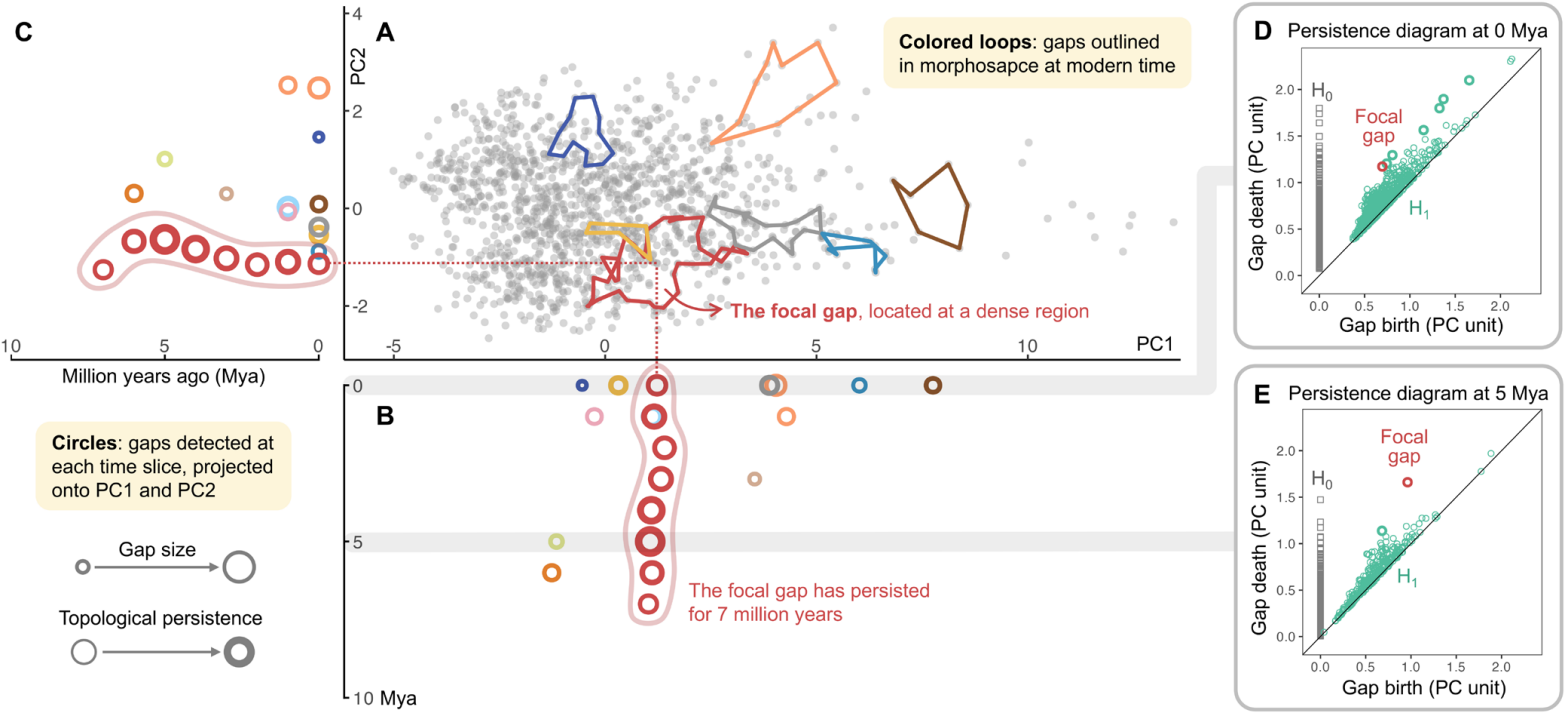
Temporal dynamics of identified gaps in Passeroidea morphospace. (A) shows extant passeroids in the morphospace formed by PC1 and PC2, with coloured loops marking notable modern gaps. Note that this is a two‐dimensional projection of a four‐dimensional space; although some loops appear folded and many data points fall within the loops, they are actually unoccupied gaps in the full 4‐D trait space. (B and C) indicate the position of detected 4‐dimensional gaps projected onto PC1 and PC2, respectively, at each time slice (in million years ago, Mya). Each circle represents a gap, with its size reflecting relative gap size (mean distance from the gap centroid to its vertices), and stroke thickness indicating topological persistence (i.e., persistence of the gap structure across distance scales in trait space). Circles with the same colours depict gap series, representing gaps located in similar regions of morphospace across consecutive time slices. One particular gap (the focal gap) has persisted for 7 million years in a relatively dense region in the morphospace. Beyond the focal gap, only one other gap lasts more than 1 million years. (D and E) show the detected topological features of two homology groups, H_0_ (connected components; grey squares) and H_1_ (loops; green circles) at the present time and at 5 Mya, respectively. This study focuses on H_1_ features (gaps), with the focal gap shown in red throughout all panels.

**FIGURE 4:**
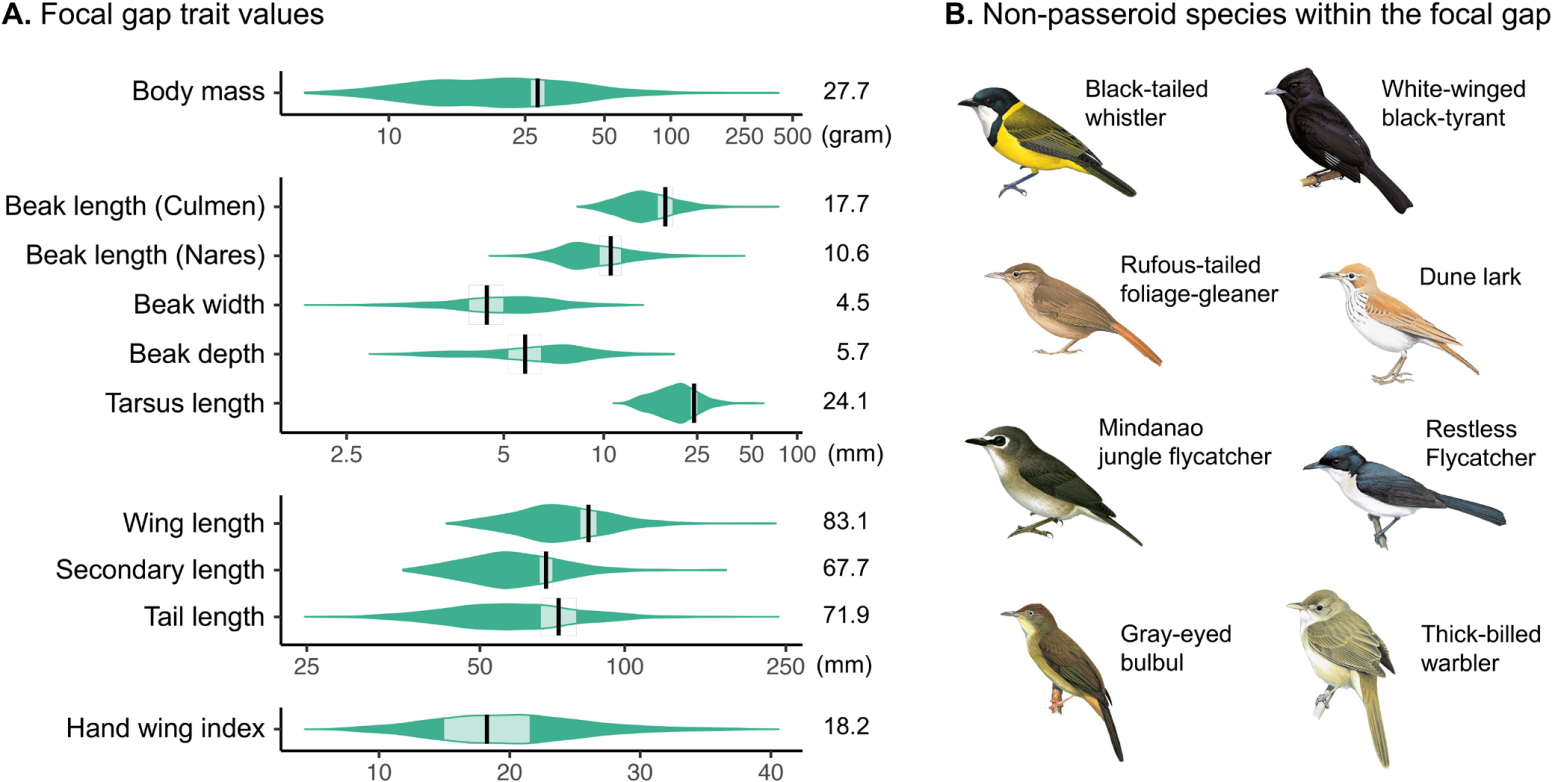
Bird morphology associated with the focal gap identified in Passeroidea morphospace. (A) Morphological trait values corresponding to the focal gap. The green violin plots show the trait distribution of all passeroid species, and black bars mark the centroid value corresponding to the focal gap. The pale shaded areas indicate the trait range associated with the gap (here defined as half the average distance from the centroid to the gap vertices). Note that because the topological structure identified in this trait space is a loop (H1 homology) rather than a solid hypervolume, the gap range represents a summary characteristic rather than the actual geometric shape of the gap. (B) Illustrations of example non‐passeroid species that occupy the focal gap region of trait space, sourced from Birds of the World, Cornell Lab of Ornithology.

One particular morphological gap (hereafter the focal gap) stood out by persisting for 7 million years and exhibited high topological persistence within each time slice (Figure 3; Table S2). This gap occurred in a densely occupied region of morphospace and corresponded morphologically to a medium‐sized songbird with a relatively thin beak (Figure 4). Despite abundant surrounding species, this trait combination remained consistently unoccupied throughout evolutionary time in the Passeroidea.

### 3.2 The Focal Gap Deviates From Stochastic Diversification Expectations

To assess whether the focal gap could result from a stochastic, undirected process, we generated 10 null trait datasets simulating Brownian motion evolution using the same phylogeny, root values and evolutionary rates as in the empirical data.

**FIGURE 5:**
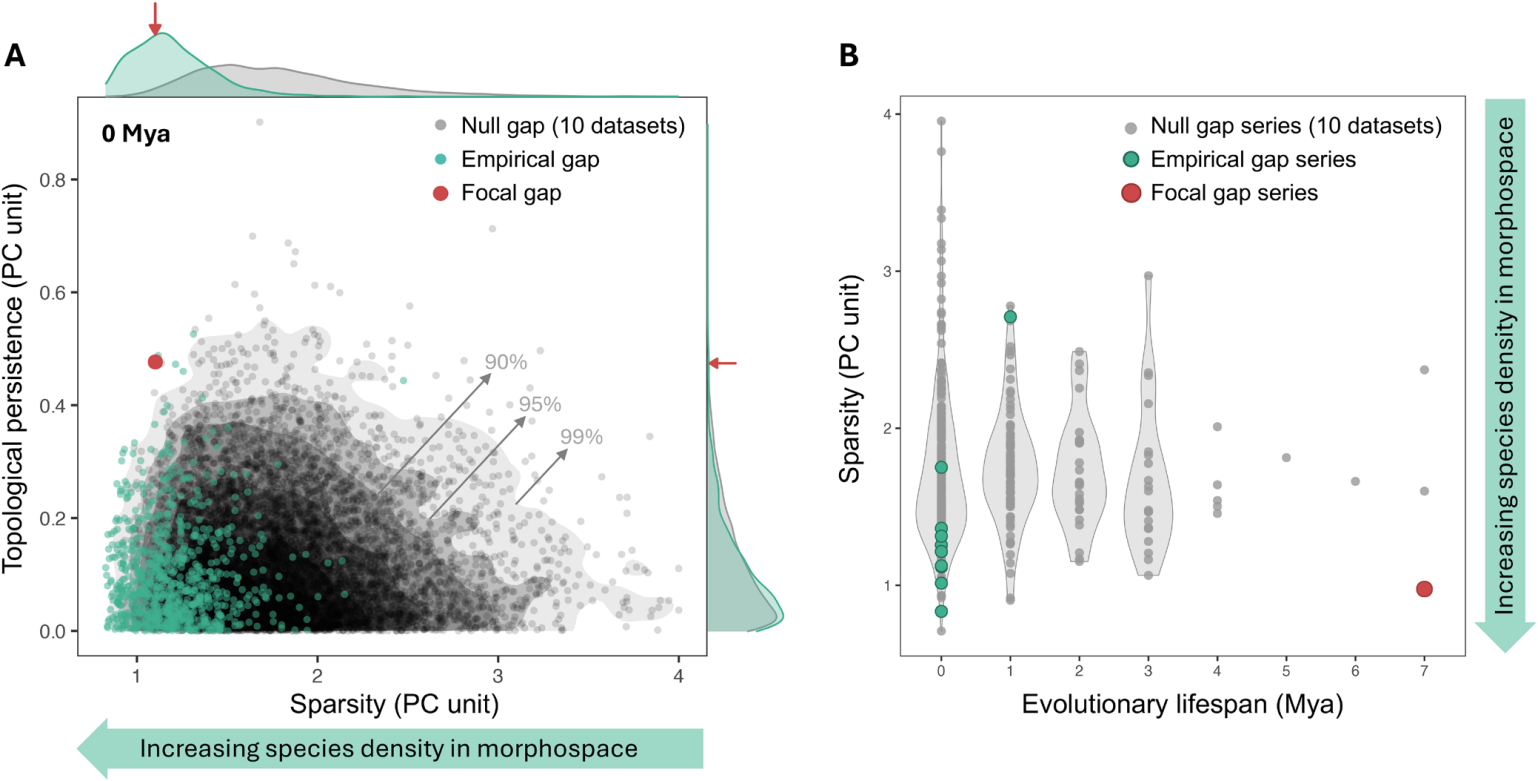
Comparison of the focal morphological gap to gaps detected in null datasets. (A) Topological persistence versus sparsity for gaps identified at the modern time. Sparsity is defined as the mean distance from the gap centroid to the nearest 5% of data points in the full trait space; lower sparsity indicates denser species distribution in the morphospace. Each point represents a gap from either the empirical dataset (green) or one of 10 null datasets simulating Brownian motion trait evolution (grey). The focal gap is shown in red. Contours indicate the 99%, 95%, and 90% quantiles of the null gap distribution. (B) Sparsity versus evolutionary lifespan of gap series, where each gap series represents evolutionarily linked gaps detected in similar morphological locations across time slices. The focal gap stands out for its combination of high topological persistence, long evolutionary lifespan (7 million years) and location within a densely occupied region of morphspace.

Compared to gaps detected in the null datasets, the focal gap was notable in that it exhibited both a long evolutionary lifespan and high topological persistence within a densely occupied region of morphospace (Figure 5). At the modern time slice, most gaps with high topological persistence in the null datasets were found in sparser regions of morphospace, whereas the focal gap was situated in a densely populated area (Figure 5A). Across time, gap series in the null datasets were typically short‐lived; only four persisted for more than 4 million years among all gap series detected across the 10 null datasets, and none of these occurred in regions as densely occupied as the focal gap (Figure 5B). These differences suggest that the focal gap is unlikely to have emerged under a stochastic model of undirected trait diversification, arguing against the stochastic diversification hypothesis (Hypothesis C).

### 3.3 No Evidence for Historical Occupation or Missing Data

We next evaluated whether the focal gap might be a remnant of previously existing but now extinct morphologies (Hypothesis D). Ancestral trait reconstructions revealed no evidence of occupation or traversal through the gap by ancestral passeroid lineages at any time slice (Figure S2). To complement the limitation that ancestral reconstructions only include ancestors of extant species, we also examined recently extinct species with available measured or imputed morphological trait data (those extinct within the past ~130,000 years). Among 60 such extinct passeroid species, only three were located near the gap, and none fell within it (Figure S3A). Collectively, these findings provide little support for the extinction hypothesis.

To test whether the gap could be a result of missing data (Hypothesis E), we examined the 40 species excluded from our analysis due to incomplete traits. Only one of their reference species (used for trait imputation in the original dataset) fell near the gap (Figure S3B), making it unlikely that the focal gap is caused by missing species clustering in the gap region.

### 3.4 Morphological Viability Outside the Passeroidea

To evaluate whether the persistent gap reflects an intrinsically infeasible morphology (Hypothesis B), we searched for species from other passerine clades that occupy the same region of morphospace. We found multiple non‐passeroid species located within the focal gap (Figure S4, Table S3), confirming that this trait combination is biologically viable. Moreover, passeroid species surrounding the gap span multiple families (Figure S4, Table S4), suggesting that the absence of this morphology is not due to a lineage‐specific constraint, but instead reflects a broader pattern across the clade. These findings argue against intrinsic constraints as the cause of the gap.

**FIGURE 6:**
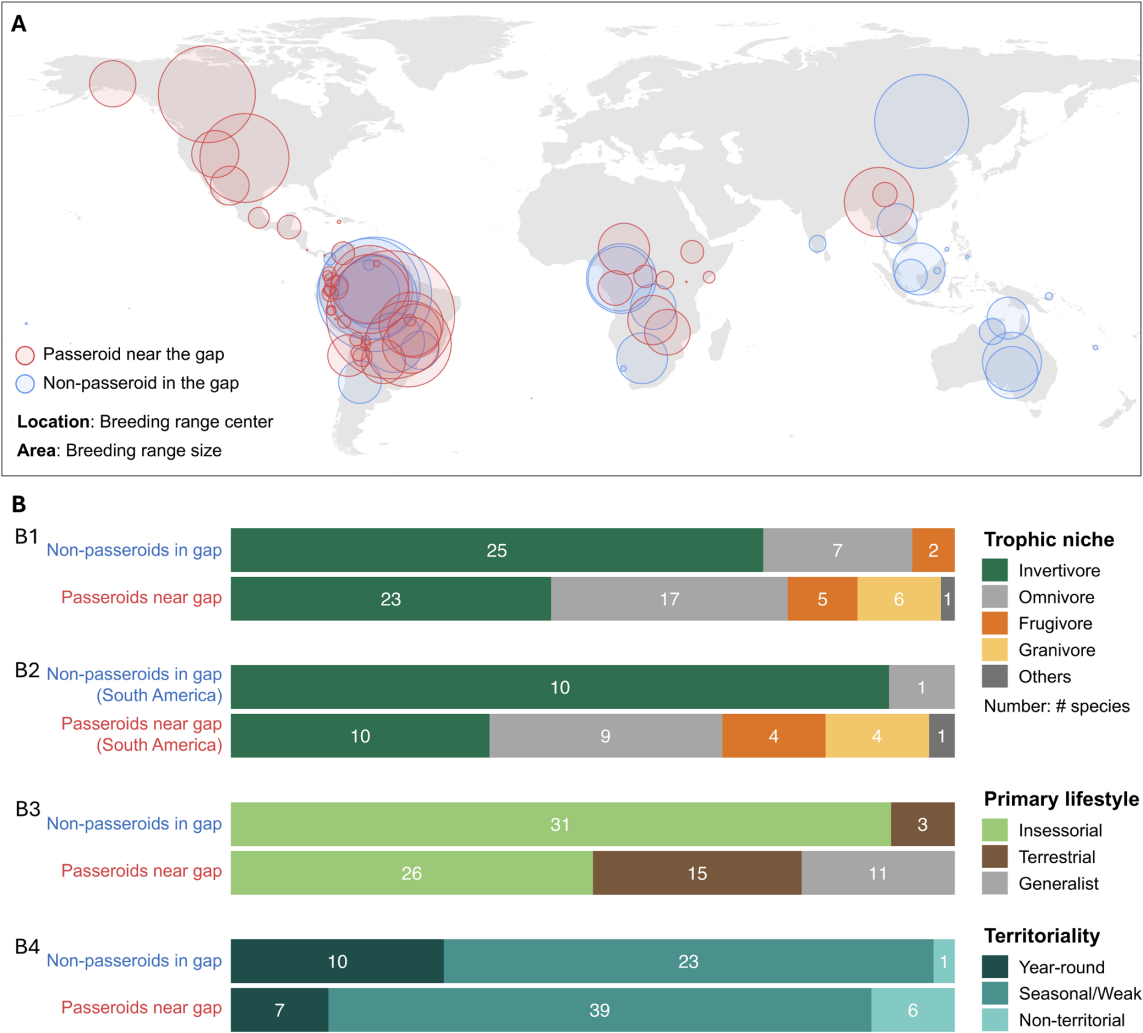
Geographical distribution and ecological traits of non‐passeroids in the gap and passeroids near the gap. (A) Global distribution of breeding range centers for non‐passeroids occupying the focal gap (blue) and passeroids near the gap (red). Circle size reflects the true breeding range area (not shape), and location indicates the geographic center of each species' breeding distribution. (B) Ecological trait profiles of non‐passeroids in the gap and passeroids near the gap. Bar plots show species counts grouped by trophic niche (B1 and B2), primary lifestyle (B3), and territoriality (B4). Trophic niche traits are summarised for all species (B1) and separately for species in South America (B2), where geographic overlap is most pronounced. The primary lifestyle “insessorial” refers to species that habitually perch above the ground, whether in vegetation or on elevated substrates such as rocks or artificial structures.

In addition, the non‐passeroid gap occupants are geographically widespread (Figure 6A), occupying biogeographic regions where passeroids also occur. This suggests that suitable ecological niches for gap morphologies exist and are accessible to Passeroidea, making the niche absence hypothesis (Hypothesis F) unlikely.

### 3.5 Competitive Exclusion as the Most Plausible Explanation

That the morphological gap persists within, but not outside, the Passeroidea raises the possibility of competitive exclusion (Hypothesis G), and three additional lines of observation provide support for this explanation. First, the non‐passeroid gap occupants are predominantly perching insectivores (Figure 6B1,B3) that generally exhibit territorial behaviour (Figure 6B4) and are primarily distributed in humid tropical or subtropical forests (Figure 6A). Their narrow niche specialisation and capability for resource defence suggest a high potential for competitive behaviours.

Second, phylogenetic and biogeographic evidence indicates that the ancestors of non‐passeroid species falling within the morphological gap colonised key habitats earlier than did those of passeroids with similar morphologies. In South America, non‐passeroid gap occupants (e.g., Tyrannidae, Tityridae, Furnariidae) arrived during the Oligocene or early Miocene (~20–30 Mya), whereas passeroids near the gap (e.g., Thraupidae, Icteridae, Passerellidae) arrived later, mostly within the last 3–10 million years following or close to the closure of the Isthmus of Panama (Oliveros et al. 2019; Weir et al. 2009). A similar pattern is seen in Africa, where the Sylviida clade (e.g., Alaudidae, Pycnonotidae) originated or arrived during the Oligocene (~25–30 Mya), preceding the arrival of passeroid lineages like the Ploceidae and Motacillidae, which have Eurasian origins and likely entered Africa only after the early Miocene (Alström et al. 2013, 2023; Oliveros et al. 2019; Voelker 1999). This historical precedence raises the possibility that early arrivals preempted available ecological niches, limiting opportunities for passeroids to evolve into the same region of trait space.

Third, in South America where the geographic overlap between the two groups is most extensive, the dietary contrast between passeroid and non‐passeroid species became more pronounced. Non‐passeroid species occupying the gap in morphospace exhibit strong niche specialization, while morphologically similar passeroids display more generalized diets (Figure 6B2), suggesting potential niche displacement of passeroids in response to competition.

Together, these findings support a scenario in which early‐colonising, ecologically specialised non‐passeroids preempted and defended a distinct ecological niche, thereby constraining passeroid lineages from evolving into that region of morphospace.

## 4 Discussion

### 4.1 Competitive Exclusion as the Most Likely Driver of the Focal Morphological Gap

By applying persistent homology to morphological trait data reconstructed through time, we identified a morphological gap within the Passeroidea morphospace—a region that has remained consistently unoccupied for approximately 7 million years based on the BirdTree phylogeny (Jetz et al. 2012). After evaluating seven hypotheses, we ruled out six, leaving competitive exclusion as the most plausible explanation. In particular, our findings support a scenario in which early‐colonising, ecologically specialised non‐passeroids preempted a viable region of morphospace, thereby limiting evolutionary access for passeroid lineages.

The morphology associated with the focal gap is realised by non‐passeroid species that are primarily insectivores foraging in vegetation in the tropical system. This pattern is consistent with the relatively slender beak shape of this morphology, which is typically associated with feeding on invertebrates, emphasising faster biting rates over greater biting force (Navalón et al. 2019). Insectivory has also been documented as an ecological strategy linked to high niche specialisation and strong interspecific aggression (Robinson and Terborgh 1995; Sherry et al. 2020). The idea that early‐colonising species can preempt ecological niches is a well‐documented phenomenon across diverse taxa (Stroud et al. 2024). In the Neotropics, such dynamics have been observed in bird communities in which early colonising Tyranni dominate in species richness, but later‐arriving Passeri exhibit greater functional diversity, likely reflecting niche displacement to avoid competition (Almeida et al. 2018). This pattern aligns with our results, where the non‐passeroid gap occupants in South America all belong to the Tyranni clade, suggesting that early colonisation and ecological specialisation may have played a central role in excluding passeroids from evolving into what has for them become a gap in morphospace.

These findings suggest that long‐term morphological gaps can arise not just from intrinsic constraints but from historical contingencies in community assembly. Consequently, disruptions to established community structures, such as environmental change and species invasions, may alter competitive dynamics, potentially opening previously inaccessible regions of morphospace or reinforcing new morphological gaps.

### 4.2 Other Morphological Gaps: Random and Non‐Random Drivers

Although the focal gap appears to be a product of long‐term competitive dynamics, other gaps identified in the Passeroidea morphospace were generally shorter‐lived, with characteristics not particularly deviating from those produced under simulated Brownian motion, suggesting they may have arisen from stochastic diversification processes.

Interestingly, compared to null datasets, gaps in the empirical dataset tended to occur in denser regions of morphospace (i.e., those with low sparsity) and were smaller in size (Figure 5, Figure S5), despite the overall similar variance on the PC axes (Figure S6). This pattern may reflect niche packing, in which closely related species differentiate to coexist in the same environment, leaving smaller gaps. Future research could test this idea by comparing gap patterns between sympatric and allopatric species assemblages, with the expectation that sympatric groups will show lower sparsity and smaller gaps.

### 4.3 Limitations and Methodological Considerations

Persistent homology is computationally intensive, especially in terms of memory usage for our particular analysis, which limits the ability to explore a broad range of evolutionary models and phylogenetic hypotheses. To maintain minimal assumptions and compatibility with a consensus tree with polytomies, we used ancestral state reconstruction under a Brownian motion model. While Brownian motion is widely used as a baseline in comparative studies, it assumes constant evolutionary rates, unconstrained trait diffusion and the absence of directional or stabilizing selection (Revell 2025). Deviations from these assumptions could influence the absolute duration or distribution of reconstructed morphological gaps. For instance, it is possible that stabilizing selection could reduce trait diffusion and lengthen gap persistence, whereas heterogeneous evolutionary rates or changing optima could produce more transient or irregular gaps. Because we modeled both empirical reconstructions and null simulations under the same Brownian motion framework, any systematic bias would likely influence both analyses similarly. Although alternative evolutionary models could alter the expected structure of null gaps and the magnitude of deviation we observe, our inference that the focal gap detected at the present day represents a departure from null expectations likely remains qualitatively robust. Even if the evolutionary lifespan of this gap were shorter under alternative evolutionary assumptions, a competitive exclusion process remains a possible explanation for its persistence at contemporary timescales.

The use of a consensus phylogeny containing unresolved polytomies could inflate the spread of ancestral traits, potentially affecting the size or frequency of detected gaps in morphospace. Ideally, such analyses could incorporate numerous fully resolved candidate dichotomous trees to account for phylogenetic uncertainty while maintaining dichotomous tree structure, but the high computational demand currently makes this impractical. Nevertheless, because both the empirical and null datasets were analysed using the same consensus tree, any biases introduced by tree topology would affect both equally. This makes it unlikely that the distinctive properties of the focal gap are an artifact of tree topology.

The estimated branching time of the Passeroidea has changed considerably over time, from close to 40 million years ago in the BirdTree phylogeny (Jetz et al. 2012) to ~18 million years ago in more recent work (Stiller et al. 2024). However, the BirdTree dataset remains the only available global phylogeny for full species‐level coverage, and thus was used in our analysis. A more recent divergence time would shift the temporal scale of our results; for example, the focal gap identified as unoccupied for 7 million years may, under updated calibrations, span closer to 3–4 million years. Nonetheless, the central finding that this gap has remained persistently unoccupied throughout the evolutionary history of the Passeroidea remains robust.

We acknowledge that our evaluation of the extinction hypothesis (Hypothesis D) is constrained by the temporal scope of available data, as the extinct species included here are relatively recent (< 130,000 years). Our conclusion is therefore based on currently available evidence and could change if older fossils representing the missing morphology are discovered. Nevertheless, even if such ancestral forms once existed, the extinction could still be consistent with the action of competitive exclusion. Moreover, even if fossils were found that shorten the estimated duration of the focal gap (currently ~7 million years), the gap would likely remain distinct from those generated under the null models because it occurs within an exceptionally dense region of morphospace (Figure 5B).

Our analysis comparing passeroid and non‐passeroid species relies on the present‐day breeding ranges to represent species distributions and to indirectly infer potential interactions over time. Although the sympatric comparison in South America includes many lineages that are largely endemic to the region (Figure 6B2; Tables S3 and S4), overall, we did not account for the (largely unknown) dynamics in species' geographic ranges across evolutionary time or for fine‐scale habitat segregation. Consequently, the degree of actual spatial overlap—and thus direct interactions—remains uncertain. Even so, the substantial contrasts in ecological traits between the two groups, particularly in the sympatric analysis, provide evidence consistent with our hypothesis of competitive exclusion. Studies on behavioural observations (e.g., Robinson and Terborgh 1995) would be valuable for further testing the hypothesis.

Finally, we acknowledge that the 10 external morphological traits used in this analysis do not capture the full range of ecological strategies employed by bird species. Furthermore, morphology and ecological niche need not always correspond directly; similar niches may be filled by species with different morphologies, and vice versa. Nevertheless, the presence of a clear gap within our trait set warrants explanation, regardless of whether other unexamined traits show similar patterns. Our topological framework provides a way to identify such patterns in trait space and to explore potential underlying mechanisms. Clarifying the specific ecological processes involved, including whether and how interspecific competition acts in vivo, will benefit from further investigation.

### 4.4 Broader Implications

This study demonstrates the application of persistent homology, a topological data analysis tool, to macroevolutionary questions by examining the structure of unoccupied regions in trait space. By tracing high‐dimensional morphological gaps through evolutionary time, we reveal robust features of trait distributions that are often overlooked by conventional dimensionality reduction approaches. This framework also allows us to distinguish short‐lived, potentially stochastic gaps from those that are both topologically persistent (stable across scales in trait space) and evolutionary persistent (stable over time).

Although our analysis focuses on the Passeroidea and continuous morphological traits, the approach is broadly applicable across taxonomic groups and trait types, including non‐continuous variables (e.g., Nguyen et al. 2022). For example, it could be used to identify functional gaps or underutilised climatic niches in plant communities to assess invasion risk, or to detect missing floral trait combinations and their potential associations with pollinator availability. By treating gaps in trait space (i.e., the absence of species with particular trait combinations) as informative biological signals, our approach provides a new perspective on why some organismal forms or strategies do not exist. This novel and generalizable framework integrates trait‐based approaches with concepts from evolutionary processes, ecological forces and biogeographic history to advance our understanding of the drivers and limits of biodiversity.

## Author Contributions

Stephanie Y. Chia, Anshuman Swain, William F. Fagan conceptualised the idea. Stephanie Y. Chia, Anshuman Swain, Nathaniel Josephs, Lizhen Lin, William F. Fagan developed methodology. Stephanie Y. Chia analysed the data and wrote the manuscript. William F. Fagan supervised the project. All authors contributed to manuscript revisions.

## Supporting information


**Figure S1:**Evolutionary trajectories of passeroid morphological traits along the four principal component axes.
**Figure S2:** Evolutionary trajectories of passeroid morphological traits projected onto PC1 and PC2.
**Figure S3:** Recently extinct and reference species of passeroids projected onto the morphospace defined by the first two PCs of the extant passeroid species included in the analysis.
**Figure S4:** Phylogenetic tree of Passeriformes showing the positions of species near or within the focal morphological gap.
**Figure S5:** Comparison of gap properties between empirical and null datasets.
**Figure S6:** Empirical and simulated null trait distributions in morphospace and their corresponding persistence diagrams.
**Table S1:** Loadings of 10 morphological traits onto the principal components for extant Passeroidea species.
**Table S2:** Summary of notable morphological gaps in passeroid morphospace across time slices.
**Table S3:** List of non‐passeroid species occupying the focal morphological gap and their ecological traits.
**Table S4:** List of passeroid species near the focal morphological gap and their ecological traits.

## Data Availability

All code used for analyses, along with the data required to reproduce the results and figures, is archived on Zenodo (https://doi.org/10.5281/zenodo.18040375).
